# A Quasi-experimental Study to Assess the Effectiveness of the Neonatal Golden Hour Management Workshop on Knowledge Among Healthcare Providers in Latifa Women and Children Hospital, Dubai

**DOI:** 10.7759/cureus.98119

**Published:** 2025-11-29

**Authors:** Khaled El-Atawi, Merlin N Kumari, Khadija M AL Sulaimi, Udayakumari Pethaperumal, Sylvia Fernandes

**Affiliations:** 1 Pediatrics/Neonatal Intensive Care Unit, Latifa Women and Children Hospital, Dubai, ARE; 2 In-service Education and Training Unit, Latifa Women and Children Hospital, Dubai, ARE; 3 Nursing Department, Latifa Women and Children Hospital, Dubai, ARE

**Keywords:** education, golden hour, healthcare score, newborn, simulation-based learning

## Abstract

Background: The first hour of life, often referred to as the "golden hour," represents a critical window for initiating timely, evidence-based interventions that stabilize the newborn and reduce the risk of complications. This period demands coordinated efforts in thermoregulation, respiratory support, glucose monitoring, and early nutrition to optimize neonatal outcomes. Despite the existence of well-established clinical guidelines, knowledge and practice gaps among healthcare providers continue to pose significant challenges. These discrepancies contribute to variations in the quality of neonatal care, particularly in high-risk settings. To address this issue, structured educational programs and targeted workshops have emerged as effective strategies to enhance healthcare providers' competencies, ensuring consistent and high-quality care during this vital phase.

Objective: This study aimed to assess the effectiveness of the neonatal golden hour management workshop on the knowledge scores of healthcare providers.

Materials and methods: A quasi-experimental quantitative research design study was conducted at Latifa Women and Children Hospital, one of the largest maternity hospitals in Dubai, consisting of all its employees. The hospital has around 600 nurses working in various maternity units and a yearly census of 4000-5000 deliveries, and all normal vaginal deliveries are conducted by obstetric nurses or midwives. Nurses from all categories in Latifa Women and Children Hospital from the months of February 2024 to November 2024 participated in the survey.

Results: The proportion of participants with good, very good, and excellent knowledge increased from 12%, 0%, and 0% in the pretest to 76%, 20%, and 4% in the posttest, respectively, indicating substantial improvement in overall knowledge levels. Participants aged 40-50 years and 35-40 years had more knowledge and higher scores (71.16±25.16 and 70.18±9.95, respectively). The participants working in the intensive care unit and emergency departments had more knowledge and higher scores (74.58±8.10 and 70.71±8.86, respectively).

Conclusion: This result highlights the crucial role of structured workshops and training courses in improving the preparedness of healthcare providers to deliver timely and effective neonatal care during the golden hour. These findings support the integration of regular, evidence-based educational interventions into hospital training policies to ensure sustained improvements in neonatal outcomes.

## Introduction

Clinical events that occur during the physiologic transition from intrauterine to extrauterine life are especially significant for the extremely low birth weight (ELBW) infant (≤1000 g) [1]. Following birth, ELBW infants are susceptible to the rapid development of hypothermia, hypoglycemia, hypotension, and respiratory failure [2,3]. Resuscitation in the delivery room (DR) and stabilization during admission to the neonatal intensive care unit (NICU) involve a series of interdependent tasks and procedures. These interventions must be performed quickly, proficiently, and systematically to minimize the short-term sequelae of prematurity, which contribute to the risk of long-term morbidity and mortality [4].

Neonatal morbidity and death are permanently impacted by care practices during the first 60 minutes of life for extremely preterm neonates [5,6]. The phrase "golden hour" refers to the crucial hour following an initial injury during which successful stabilization increases survival. It originated from the out-of-hospital stabilization of adult trauma patients. The first hour of newborn care following delivery has recently been referred to by this phrase [7]. The golden hour refers to a potentially standardized, evidence-based approach to DR care, communication, and teamwork for very preterm infants (less than 32 weeks' gestational age (GA)), with the goal of improving outcomes. However, the precise procedures included or recommended within the neonatal golden hour are currently unclear [8]. Maintaining normothermia, targeting oxygen saturation appropriately, using non-invasive respiratory support on a regular basis, and preventing hypoglycemia are among the goals. There are no precise criteria for the timing of interventions within the first hour of life, despite the fact that international resuscitation guidelines concentrate on standardized DR stabilization [9].

Critical to the success and sustainability of any golden hour initiative is the recognition of the continuous educational process involving multidisciplinary team collaboration to ensure coordination between providers in the DR and beyond [10]. Standardization of practices in the care of extremely premature neonates during the first hour of life leads to improved outcomes. A coordinated focus on the golden hour in ELBW neonates has been shown to improve short-term and long-term outcomes [11].

Simulation-based learning (SBL) has been shown to effectively improve medical knowledge, procedural proficiency, comfort with undertaking taught tasks, inter-professional communication, teamwork, and teaching skills [12,13]. This study aimed to find the effectiveness of the neonatal golden hour management workshop on knowledge among nurses in the selected government hospital. Thus, nurses who attended the workshop will gain enough knowledge and confidence to face the real situation and act appropriately to save the patient at the earliest by implementing learned advanced interventions.

Research hypothesis

H1

There is a significant difference in pre- and posttest knowledge scores of healthcare providers who attended the golden hour management workshop. 

H0

There is no significant difference in pre- and posttest knowledge scores of healthcare providers who attended the golden hour management workshop. 

H1

There is a significant association between the selected demographic variables and knowledge scores of healthcare providers who attended the golden hour management workshop. 

H0

There is no significant association between the selected demographic variables and knowledge scores of healthcare providers who attended the golden hour management workshop.

## Materials and methods

Study design and setting

A quasi-experimental quantitative research design study was conducted at Latifa Women and Children Hospital, one of the largest maternity hospitals in Dubai, consisting of all its employees. The hospital has around 600 nurses working in various maternity units and a yearly census of 4000-5000 deliveries, and all normal vaginal deliveries are conducted by obstetric nurses or midwives. All categories of nurses working who attended the workshop from February 2024 to November 2024 were included as sample.

Sample size and selection

Sampling is the process of selecting a portion of the population to represent the entire population. Purposive sampling techniques were used to select the sample. One hundred fifty samples were included in the study.

Sample selection criteria

In the current study, we included the nurses who attended the golden hour workshop and were willing to participate. However, we excluded the nurses who attended the golden hour workshop before the study period.

Collection of data

A structured questionnaire was prepared based on the 10 components of the golden hour [14,15], namely, antenatal counseling and team pre-briefing, delayed cord clamping, support to the respiratory system, support to the cardiovascular system, prevention of hypoglycemia-initiation of breastfeeding, infection prevention/laboratory investigation/arterial blood gas (ABG) analysis, temperature control, monitoring/record-code narrator documentation, teamwork/de-briefing/communication and family counseling, and emotional support. From each component, two questions are being prepared and sent for expert validity, and data were collected by sending an online questionnaire to the study participants.

A structured questionnaire was prepared based on the components of golden hour [14,15].

Validity of the tool

To determine the content validity of the data collection tool, the prepared instrument along with the proposal was sent to five experts. The experts were requested to give their opinion on the appropriateness and relevance of standardized tools. Modifications were made according to the experts' opinion.

Ethical considerations

Ethical permission was obtained from the Dubai Scientific Research Ethics Committee (DSREC) of the Dubai Health Authority (approval number: DSREC-11/2024_37). The study was conducted after obtaining written informed consent from all the participants. Assurance was given to all the participants that confidentiality will be maintained throughout the study.

Pilot study

A pilot study is a test run of the research to confirm its feasibility and conduct the real study based on the test run changes. A pilot study was conducted among 10 samples who met the inclusion criteria.

Data collection

The purpose and significance of the study were communicated to the participants in advance, and the staff who came to the workshop were given a pretest and posttest to assess their knowledge. The study subjects were allowed to withdraw consent at any time. The researchers remained neutral in collecting the data by sending the questionnaire via Microsoft Forms (Microsoft Corp., Redmond, WA, USA) to the study participants.

Data analysis methods

Data was analyzed based on descriptive and inferential statistics. Statistical analysis was done using IBM SPSS Statistics for Windows, V. 26.0 (IBM Corp., Armonk, NY, USA). Quantitative variables were presented as mean and standard deviation (SD) and were analyzed by an unpaired Student's t-test. Qualitative variables were presented as frequency and percentage (%) and were compared by the chi-squared test. Fisher's exact test or Monte Carlo correction was used to correct for chi-square when more than 20% of the cells have expected count less than 5. The analysis of variance (ANOVA) F-test is used to compare the quantitative variables between more than two groups of normally distributed data. The paired t-test is used to test the mean difference between pairs of measurements. A p-value of <0.001 was considered statistically highly significant.

## Results

**Figure 1:**
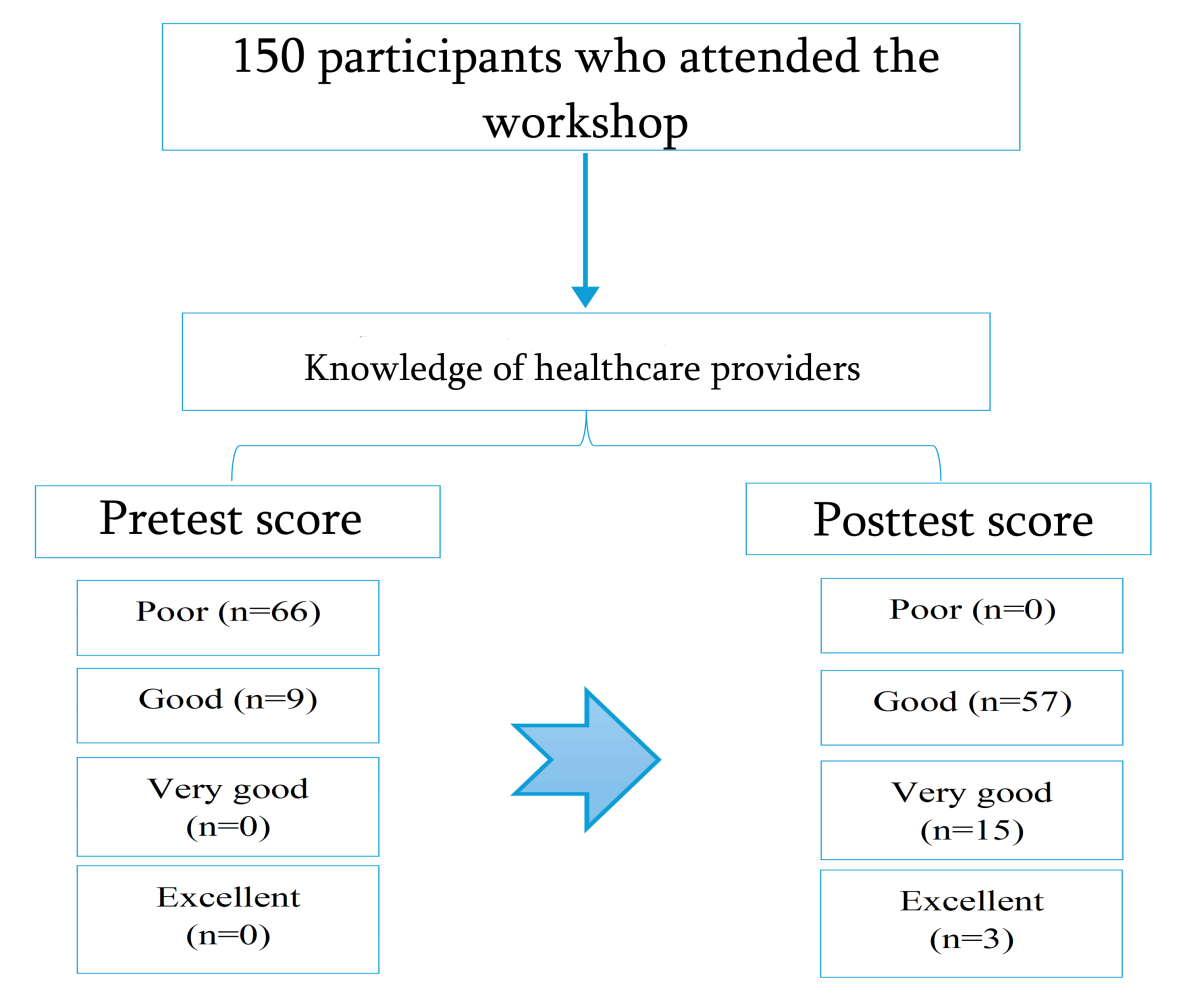
Flowchart of the participants studied.

A flowchart illustrates that there were 150 participants who attended the workshop and their knowledge was assessed using a pretest and posttest at Latifa Women and Children Hospital, one of the largest maternity hospitals in Dubai (Figure 1). Participants were categorized into four groups based on their pretest and posttest scores: 66 (44%) demonstrated poor knowledge, 57 (44%) had good knowledge, 15 (10%) had very good knowledge, and 3 (2%) had excellent knowledge.

The findings of the study revealed a highly significant improvement in participants' knowledge following the golden hour management workshop, as indicated by the posttest scores (p<0.001). Post-intervention, 76% of participants achieved good knowledge, 20% demonstrated very good knowledge, and 4% reached excellent knowledge levels compared to only 12%, 0%, and 0%, respectively, in the pretest (Table 1, Figure 1).

**Table 1:** Knowledge of healthcare providers: posttest score versus pretest score. *Statistically significant at p≤0.05 P: p-value for comparing the two studied groups; χ^2^: chi-squared test; MC: Monte Carlo

Score interpretation	Pretest score (N=75)	Posttest score (N=75)	Total (N=150)	χ^2^	MC (P)
Poor (<70%)	66 (88)	0	66 (44)	94.573	<0.001*
Good (70-80%)	9 (12)	57 (76)	66 (44)		
Very good (80-90%)	0	15 (20)	15 (10)		
Excellent (90-100%)	0	3 (4)	3 (2)		
Total	75	75	150	-	-

**Figure 2:**
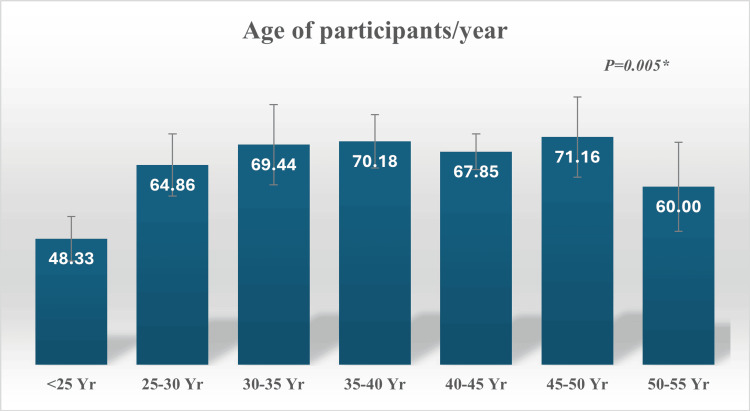
Relation between healthcare providers' knowledge scores and ages.

In the current study, a significant association was observed between participants' knowledge scores and their age (p=0.005). Participants aged 40-50 years and 35-40 years demonstrated higher knowledge scores (71.16±25.16 and 70.18±9.95, respectively). Conversely, participants younger than 25 years recorded the lowest knowledge scores (48.33±21.50) (Figure 2).

With respect to different units, participants working in the intensive care unit (ICU) and emergency department demonstrated the highest knowledge scores (74.58±8.10 and 70.71±8.86, respectively), followed by those in the delivery suite (68.27±11.04). In contrast, participants from the operation theatre reported the lowest knowledge scores (60.00±20.31). However, no statistically significant relationship was found between knowledge scores across the various departments, including maternity, operation theatre, delivery suite, NICU, ICU, and emergency (p>0.05) (Table 2).

**Table 2:** Relation between healthcare providers' knowledge scores and units. Statistically significant at p≤0.05 χ^2^: chi-squared test; P: p-value for comparing the two studied groups; NICU: neonatal intensive care unit, ICU: intensive care unit

	Healthcare providers' knowledge scores				Mean±SD	χ^2^	P
	Poor (<70%)	Good (70-80%)	Very good (80-90%)	Excellent (90-100%)			
Maternity	24 (36.36)	14 (21.2)	5 (33.3)	3 (100)	65.97±17.18	1.396	0.926
Operation theatre	3 (4.5)	2 (3.03)	0	0	60.00±20.31		
Delivery suite	12 (18.2)	15 (22.7)	2 (13.3)	0	68.27±11.04		
NICU	23 (34.8)	22 (33.3)	6 (40)	0	63.82±18.01		
ICU	2 (3.03)	8 (12.1)	2 (13.3)	0	74.58±8.10		
Emergency	2 (3.03)	5 (7.6)	0	0	70.71±8.86		
Total	66	66	15	3	66.40±15.83	-	-

Similarly, no significant association was observed between participants' knowledge scores and years of experience (p>0.05) (Table 3).

**Table 3:** Relation between healthcare providers' knowledge scores and years of experience. *Statistically significant at p≤0.05. χ^2^: chi-squared test; P: p-value for comparing the two studied groups

	Healthcare providers' knowledge scores				Mean±SD	χ^2^	P
	Poor (<70%)	Good (70-80%)	Very good (80-90%)	Excellent (90-100%)			
<5 years	5 (7.6)	6 (9.1)	3 (20)	1 (33.3)	67.33±21.62	14.657	0.014*
5-10 years	14 (21.1)	7 (10.6)	3 (20)	1 (33.3)	62.60±19.21		
10-15 years	23 (34.8)	19 (28.8)	4 (26.7)	1 (33.3)	65.21±15.88		
15-20 years	13 (19.7)	25 (37.9)	4 (26.7)	0	70.24±11.89		
20-25 years	6 (9.1)	7 (10.6)	1 (6.7)	0	67.86±11.55		
>25 years	5 (7.6)	2 (3)	0	0	60.00±15.55		
Total	66	66	15	3	66.40±15.83	-	-

Additionally, healthcare providers' knowledge scores increased among participants who had a bachelor's education (66.54±15.92); however, there was no significant relation between healthcare providers' knowledge scores and highest education (p>0.05) (Table 4).

**Table 4:** Relation between healthcare providers' knowledge scores and highest education. Statistically significant at p≤0.05 χ^2^: chi-squared test; P: p-value for comparing the two studied groups

	Healthcare providers' knowledge scores				Mean±SD	χ^2^	P
	Poor (<70%)	Good (70-80%)	Very good (80-90%)	Excellent (90-100%)			
Bachelor	63 (95.5)	65 (98.5)	15 (100)	3 (100)	66.54±15.92	2.107	0.910
Diploma	1 (1.5)	0	0	0	60.00±0.000		
Master	2 (3.03)	1 (1.5)	0	0	61.67±16.07		
Total	66	66	15	3	66.40±15.83	-	-

In contrast, there was a highly significant relation between healthcare providers' knowledge scores and courses attended previously (p>0.001). The participants who previously attended neonatal nursing courses and other courses related to the neonatal specialty had higher scores (78.75±4.79 and 83.00±2.74) (Table 5).

**Table 5:** Relation between healthcare providers' knowledge scores and courses attended previously. *Statistically significant at p≤0.05 χ^2^: chi-squared test; P: p-value for comparing the two studied groups

	Healthcare providers' knowledge scores				Mean±SD	χ^2 ^	P
	Poor (<70%)	Good (70-80%)	Very good (80-90%)	Excellent (90-100%)			
No	61 (92.4)	9 (13.6)	0	0	53.36±12.59	11.85	<0.001*
Yes	5 (7.6)	41 (62.1)	11 (73.3)	3 (100)	77.58±8.00		
Golden hour workshop	0	2 (3.03)	0	0	75.00±0.00		
Neonatal nursing course	0	3 (4.5)	1 (6.7)	0	78.75±4.79		
Stable	0	7 (10.6)	0	0	76.43±2.44		
Others related to the neonatal specialty	0	4 (6.06)	3 (20)	0	83.00±2.74		
Total	66	66	15	3	66.40±15.83	-	-

## Discussion

The neonatal period represents a highly vulnerable stage, with the first 60 minutes after birth often referred to as the "golden hour" being particularly critical for both survival and long-term health outcomes [16]. Evidence-based interventions during this period, including thermoregulation, respiratory support, vascular access, and timely administration of medications, have consistently demonstrated improvements in both short-term outcomes (such as reduced hypothermia and hypoglycemia) and long-term outcomes (including lower risks of bronchopulmonary dysplasia, intraventricular hemorrhage, and retinopathy of prematurity), especially among extremely premature and ELBW infants [11]. Hence, this study aimed to assess the effectiveness of neonatal golden hour management on the knowledge scores of healthcare providers before and after attending the workshop. 

In our study, 76%, 20%, and 4% of participants demonstrated good, very good, and excellent healthcare knowledge regarding golden hour management, as measured by posttest scores. These findings are consistent with those of Moninder and Gopal [17], who assessed the effectiveness of a structured teaching program on the management of neonatal asphyxia among staff nurses in pediatric allied units in selected hospitals of Punjab. Their study revealed that, in the pretest, 22 (73.34%) staff nurses in the experimental group had average knowledge and eight (26.66%) had below-average knowledge. In the control group, 20 (66.67%) had average knowledge, eight (26.66%) had below-average knowledge, and only two (6.67%) demonstrated good knowledge, with no staff nurse in either group achieving excellent knowledge. Following the intervention, however, the experimental group showed notable improvement, with 80% of nurses attaining good knowledge, 13.33% achieving excellent knowledge, and 6.67% remaining at an average level. Conversely, the control group continued to exhibit lower outcomes, with 76.67% at average knowledge and 23.33% at below-average knowledge. The consistency between their results and the current study highlights that structured educational interventions are effective in enhancing knowledge among healthcare workers involved in neonatal care.

The comparison between their findings and this study underscores the effectiveness of structured educational interventions in enhancing knowledge among healthcare professionals involved in neonatal care. Similar results were observed by Bajracharya et al. [18] in a quasi-experimental study assessing the effect of educational interventions on neonatal resuscitation knowledge and skills among nursing students. That study reported a significant increase in median knowledge and skill scores post-intervention. Notably, while the intervention and control groups did not differ significantly before the training, a statistically significant difference emerged after the intervention, highlighting the substantial impact of structured training programs on competency development.

Our outcome reflects a high level of post-intervention knowledge compared with previous studies conducted in similar contexts. For instance, Kamath-Rayne et al. [19] reported a lower mean pretest knowledge score of 7.22 among Indian nurses prior to neonatal resuscitation training, while Kc et al. [20] found similarly low pretest scores in Nepal before participants received a three-day neonatal resuscitation course. These findings suggest that baseline knowledge among healthcare workers often remains limited before structured educational programs are introduced. Consistent with the present study, research in Ethiopia by Hoban et al. [21] demonstrated a substantial improvement in healthcare workers' neonatal resuscitation knowledge immediately after training, effectively closing the pre-existing knowledge gap. Likewise, Sendo et al. [22] on Ethiopian undergraduate midwifery students reported a significant rise in posttest knowledge scores among participants following the Helping Babies Breathe intervention, with all participants attaining adequate knowledge levels (p<0.001). The convergence of findings across these studies supports the conclusion that targeted, skill-based educational programs are highly effective in enhancing neonatal care knowledge. However, variations in knowledge levels across studies may be attributed to differences in course duration, content specificity, and participants' prior clinical exposure. Collectively, the evidence underscores the value of structured training interventions in improving healthcare providers' competency in critical neonatal care, particularly during the golden hour and resuscitation period.

Although the general trend across all three studies confirms the effectiveness of educational interventions in neonatal care, differences emerge in the degree of improvement achieved. The current study showed a complete elimination of poor knowledge levels in the posttest, while in Moninder and Gopal's research, some participants in the control group continued to have below-average knowledge even after the teaching program [17]. This may be explained by the difference in methodology, as the present study used a workshop-based training model that provided hands-on engagement, whereas Moninder and Gopal utilized a structured teaching program that may have been more theoretical [17]. Furthermore, differences in the study populations could also contribute to variations in outcomes. The present study focused on healthcare providers at Latifa Women and Children Hospital, Dubai, while Moninder and Gopal assessed staff nurses in pediatric allied units in Punjab, and Bajracharya et al. evaluated nursing students [17,18]. These groups vary in terms of clinical exposure, prior knowledge, and direct responsibility for neonatal care, which may influence how effectively they benefit from educational interventions. Despite these variations, the overall agreement among the studies strongly supports that structured teaching, workshops, and training interventions significantly enhance knowledge and skills in neonatal care, though tailoring the method to the characteristics of the target population may optimize the outcomes.

The present study showed that there was no significant relation between healthcare providers' knowledge scores and years of experience, and, similarly, no significant relation between healthcare providers' knowledge scores and highest educational level, even though most of the nurses in the study sample held a bachelor's degree. The current results stand in contrast to the earlier work of Maarouf [23]. In an assessment of nurses' performance for traumatic head injury patients during the golden hour, Maarouf found that a high proportion (nearly two-thirds) of nurses had unsatisfactory practices. Furthermore, that study identified a statistically significant relationship between practice levels and years of experience, as well as a highly significant relationship with nurse age. According to Maarouf, increasing age and years of experience contributed to performance improvements until the level of automatism was reached [23]. This view was supported by Delucia et al. [24] and Dale et al. [25], who confirmed that work experience influences nurses' performance. On the other hand, Collins [26] reported that total nurses' practice regarding nursing management of trauma patients in the emergency unit was satisfactory, while Browne and Merrill [27] clarified that most studied nurses demonstrated satisfactory practice regarding the assessment and management of severe musculoskeletal injury. Furthermore, the results of the current study are not in line with Gidam and Abdelgair [28], who found that years of experience strongly affected performance, with more than half of the respondents having less than one year of experience. Their results align with previous research indicating a positive association between healthcare providers' years of experience and their neonatal resuscitation performance [29-31]. The discrepancy between these studies and the present findings may be explained by methodological differences, as the current study measured knowledge while others primarily assessed practice. It may also be related to contextual factors. In the present study, most nurses were bachelor's degree holders working in a tertiary hospital in Dubai, while Maarouf [23] and Elsayed [32] studied nurses in Ain Shams University Hospitals, Collins [26] evaluated emergency nurses, and Gidam and Abdelgair [28] focused on nurses and midwives in Sudan. Regarding education, the present study differs from Elsayed [32] and Ahmed et al. [33] who both found that most nurses in Ain Shams University were diploma graduates. However, the current results are in partial agreement with Maarouf [23], who found that nearly half of the emergency nurses in her study held a bachelor’s degree. Additionally, Ahmed et al. [33] reported that nearly three-quarters of their sample studied had received a training course about trauma patients, with more than four-fifths of them gaining benefits, which agrees with Metwaly et al. [34], who found that more than half of the nurses in their study at Zagazig University had training courses about critically ill patients. Overall, these comparisons suggest that while structured training programs consistently improve performance and knowledge, the impact of years of experience and educational level is less uniform and may depend on the setting, the characteristics of the studied population, and whether outcomes are measured as knowledge or practice.

Participants who had previously attended neonatal nursing courses and other courses related to the neonatal specialty achieved significantly higher knowledge scores, indicating a strong correlation between prior training and knowledge levels. These results emphasize the direct impact of prior training on improving knowledge in neonatal care. Similar findings were reported by Gidam and Abdelgair [28] who found that 53.3% of respondents had received training, while 46.7% had not, despite receiving basic neonatal cardiopulmonary resuscitation, and their results indicated that training had a direct effect on performance with respect to basic neonatal cardiopulmonary resuscitation. Furthermore, they noted that post‐educational program knowledge of respondents regarding advanced newborn cardiopulmonary resuscitation reached 65.3% compared to the pre‐educational program, which reflected high motivation to acquire knowledge after training. However, contradictory evidence was found in studies from Baghdad and Nigeria, where no statistically significant association was observed between nurses' practices and their number of training sessions (Nour [35] and Khalid et al. [36]), suggesting that the effectiveness of training may also depend on its structure, quality, and follow-up mechanisms. In a similar vein, Bajracharya et al. [18] in a quasi-experimental study on the effect of educational intervention on neonatal resuscitation knowledge and skills among nursing students found that training provided essential knowledge and skills, enabling students to handle neonatal emergencies more effectively. Their study showed that while the median pretest knowledge scores of the intervention and control groups were not significantly different, the posttest scores became significantly different (p<0.001). The median knowledge score in the intervention group increased markedly from 30.0 to 49.0, while that of the control group only increased slightly from 29.0 to 31.5. A significant increase was noted in both groups, but the difference was larger in the intervention group (19.0) compared to the control group (2.5). Bajracharya et al. suggested that the increase in the control group's posttest knowledge could have been due to sensitization, as the initial pretest assessment may have motivated them to study neonatal resuscitation independently [18]. Their findings align with those of Pawase [37], Guleri [38], and Sarvan and Efe [39], who all demonstrated increased knowledge after educational interventions, but differ from Kumar and Patidar [40] who observed a significant rise in the intervention group only, with no corresponding improvement in the control group. Beyond knowledge, Bajracharya et al. [18] also demonstrated that educational interventions improved skills, without a significant difference, but median posttest scores showed a highly significant difference (p<0.001). This result is supported by Sarvan and Efe [39] who similarly reported a difference in favor of the intervention group in enhancing skills.

Similarly, the study conducted by Mohamed and Alatroshi [41] reported a substantial improvement in nurses' knowledge following an educational intervention on neonatal sepsis. Their quasi-experimental study showed that participants in the experimental group achieved markedly higher posttest scores compared to pretest results, indicating the effectiveness of targeted educational programs in improving clinical knowledge. Both studies, therefore, agree that structured and focused educational interventions play a crucial role in enhancing healthcare providers' competence in neonatal care. However, while the study by Mohamed and Alatroshi concentrated on neonatal sepsis management, the current research emphasized comprehensive golden hour management, which involves a broader scope of neonatal stabilization practices [41]. The difference in focus may explain variations in the specific domains of knowledge gained. Moreover, the consistent improvement across both studies supports the premise that continuous professional development and participation in specialized workshops are essential for maintaining and advancing clinical proficiency among neonatal healthcare providers.

Finally, taken together, the findings of the present study and previous research strongly affirm that participation in training and courses has a powerful influence on improving both knowledge and skills in neonatal care, with consistent evidence across diverse contexts including Dubai, Sudan, South Asia, and Africa. At the same time, the variability observed in some studies may be explained by differences in training design, follow-up evaluation, and participant motivation, highlighting the need for structured, interactive, and contextually adapted educational programs to optimize outcomes.

## Conclusions

Results of the current study concluded that there is a highly significant improvement in healthcare knowledge regarding golden hour management when comparing the participants' posttest scores vs their pretest scores. This result highlights the crucial role of structured workshops and training courses in improving the preparedness of healthcare providers to deliver timely and effective neonatal care during the golden hour. These findings support the integration of regular, evidence-based educational interventions into hospital training policies to ensure sustained improvements in neonatal outcomes.

## References

[1] Ashmeade TL, Haubner L, Collins S, Miladinovic B, Fugate K (2016). Outcomes of a neonatal golden hour implementation project. Am J Med Qual.

[2] Harriman TL, Carter B, Dail RB, Stowell KE, Zukowsky K (2018). Golden hour protocol for preterm infants: a quality improvement project. Adv Neonatal Care.

[3] Peleg B, Globus O, Granot M (2019). "Golden hour" quality improvement intervention and short-term outcome among preterm infants. J Perinatol.

[4] Croop SE, Thoyre SM, Aliaga S, McCaffrey MJ, Peter-Wohl S (2020). The golden hour: a quality improvement initiative for extremely premature infants in the neonatal intensive care unit. J Perinatol.

[5] Laptook AR, Bell EF, Shankaran S (2018). Admission temperature and associated mortality and morbidity among moderately and extremely preterm infants. J Pediatr.

[6] Tay VY, Bolisetty S, Bajuk B, Lui K, Smyth J (2019). Admission temperature and hospital outcomes in extremely preterm infants. J Paediatr Child Health.

[7] Rogers FB, Rittenhouse KJ, Gross BW (2015). The golden hour in trauma: dogma or medical folklore?. Injury.

[8] Sheng L, Zhong G, Xing R, Yan X, Cui H, Yu Z (2024). Quality improvement in the golden hour for premature infants: a scoping review. BMC Pediatr.

[9] Hodgson KA, Owen LS, Lui K, Shah V (2021). Neonatal golden hour: a survey of Australian and New Zealand neonatal network units' early stabilisation practices for very preterm infants. J Paediatr Child Health.

[10] Ardern J, Hayward B, Vandal AC, Martin-Babin M, Coomarasamy C, McKinlay C (2023). Improving golden hour care coordination: using defined roles to improve nurse confidence and care coordination of neonates following admission. J Perinat Neonatal Nurs.

[11] Lamary M, Bertoni CB, Schwabenbauer K, Ibrahim J (2023). Neonatal golden hour: a review of current best practices and available evidence. Curr Opin Pediatr.

[12] Tjeng R, Vitória P, Lito P (2025). Enhancing patient care through interprofessional education and simulation-based learning. Technological Approaches to Medical and Pharmaceutical Education.

[13] Turatsinze S, Willson A, Sessions H, Cartledge PT (2020). Medical student satisfaction and confidence in simulation-based learning in Rwanda - pre and post-simulation survey research. Afr J Emerg Med.

[14] Sharma D (2017). Golden hour of neonatal life: need of the hour. Matern Health Neonatol Perinatol.

[15] Sharma D (2017). Golden 60 minutes of newborn's life: part 1: preterm neonate. J Matern Fetal Neonatal Med.

[16] Fathi O, Bapat R, Shepherd EG, Logan JW (2018). Golden hours: an approach to postnatal stabilization and improving outcomes. Neonatal Medicine.

[17] Moninder K, Gopal CS (2017). A quasi experimental study to assess the effectiveness of structured teaching programme on knowledge regarding management of neonatal asphyxia among staff nurses at pediatric allied units in selected hospitals of Punjab. Int J Appl Res.

[18] Bajracharya S, Karki B, Shakya S, Ban RK, Shrestha N, Nagarkoti L (2025). Effect of educational intervention on neonatal resuscitation knowledge and skill: a quasi-experimental study among nursing students. Europasian J Med Sci.

[19] Kamath-Rayne BD, Thukral A, Visick MK (2018). Helping babies breathe, second edition: a model for strengthening educational programs to increase global newborn survival. Glob Health Sci Pract.

[20] Kc A, Wrammert J, Nelin V, Clark RB, Ewald U, Peterson S, Målqvist M (2017). Evaluation of Helping Babies Breathe Quality Improvement Cycle (HBB-QIC) on retention of neonatal resuscitation skills six months after training in Nepal. BMC Pediatr.

[21] Hoban R, Bucher S, Neuman I, Chen M, Tesfaye N, Spector JM (2013). 'Helping babies breathe' training in sub-Saharan Africa: educational impact and learner impressions. J Trop Pediatr.

[22] Sendo EG, Teshome GS, Jirata WK, Gebrewold LA, Gemechu RE (2025). Effect of "helping babies breathe" training on Ethiopian undergraduate midwifery students' knowledge and skills in neonatal resuscitation at public universities: a non-randomized quasi-experimental study. SAGE Open.

[23] Maarouf DM (2012). Nurses' Performance for Patients With Traumatic Head Injury During Golden Hour. https://research.asu.edu.eg/bitstream/12345678/46856/1/106620p4017.pdf.

[24] DeLucia PR, Ott TE, Palmieri PA (2009). Performance in nursing. Rev Hum Factors Ergon.

[25] Dale J, Green J, Reid F, Glucksman E, Higgs R (1995). Primary care in the accident and emergency department: II. Comparison of general practitioners and hospital doctors. BMJ.

[26] Collins ST (2008). Emergency medical support units to critical care transport teams in Iraq. Crit Care Nurs Clin North Am.

[27] Browne KL, Merrill E (2015). Musculoskeletal management matters: principles of assessment and triage for the nurse practitioner. J Nurse Pract.

[28] Gidam NN, Abdelgair WI (2023). Quasi-experimental quantitative study of training programme for nurses and midwives regarding provision of neonatal resuscitation in selected governmental hospital, (Sudan), 2018. Nurs Open.

[29] Kim YM, Ansari N, Kols A (2013). Assessing the capacity for newborn resuscitation and factors associated with providers' knowledge and skills: a cross-sectional study in Afghanistan. BMC Pediatr.

[30] Shikuku DN, Milimo B, Ayebare E, Gisore P, Nalwadda G (2017). Quality of care during neonatal resuscitation in Kakamega County General Hospital, Kenya: a direct observation study. Biomed Res Int.

[31] Wolde HF, Gonete KA, Akalu TY, Baraki AG, Lakew AM (2019). Factors affecting neonatal mortality in the general population: evidence from the 2016 Ethiopian Demographic and Health Survey (EDHS)-multilevel analysis. BMC Res Notes.

[32] Elsayed WM (2009). Effect of an Educational Program on Nurses' Performance During the Golden Hour of Care for Traumatized Patients.

[33] Ahmed SH, Taha NM, Zatton HK (2017). Nurses' knowledge and practice of trauma patients during golden hours of care. Zagazig Nurs J.

[34] Metwaly EA, Mohammed EH, Mohammed MA (2013). Nurses' performance regarding nasogastric tube feeding in intensive care units. Zagazig Nurs J.

[35] Nour NM (2012). Premature delivery and the millennium development goal. Rev Obstet Gynecol.

[36] Khalid N, Ahmad M, Tahir A, Mahmood H, Saleem S, Saleem S (2015). Basic neonatal resuscitation, knowledge assessment at primary health care centers of district Sheikhupura in Pakistan - a cross-sectional study. J Pak Med Assoc.

[37] Pawase MA (2020). A study to assess the effectiveness of training programme on knowledge and skills regarding newborn resuscitation among student nurses (3rd year BSc) at Sdmions Dharwad. Int J Sci Res.

[38] Guleri S (2022). A quasi-experimental study to assess the effectiveness of video assisted teaching programme on knowledge regarding the neonatal resuscitation in the management of birth asphyxia among GNM 2nd year students of selected nursing college of district Kangra Himachal Pradesh. Int J Appl Res.

[39] Sarvan S, Efe E (2022). The effect of neonatal resuscitation training based on a serious game simulation method on nursing students' knowledge, skills, satisfaction and self-confidence levels: a randomized controlled trial. Nurse Educ Today.

[40] Kumar N, Patidar D (2019). Assess the effectiveness of computer assisted teaching (CAT) on knowledge gain about GCS with coma patient among B.Sc. Nursing 3rd year students of selected nursing colleges at Bhopal, Madhya Pradesh, India. Trends Nurs Adm Educ.

[41] Mohamed DA, Alatroshi AM (2022). Effectiveness of an educational program on nurses' knowledge regarding neonatal sepsis: a quasi-experimental study. Med J Babylon.

